# Renal Trauma in the West of Ireland — A Regional Review

**DOI:** 10.1100/tsw.2009.22

**Published:** 2009-02-28

**Authors:** O. A. Raheem, M. S. Floyd, R. G. Casey, I. M. Cullen, M. O. Corcoran, H. C. Bredin, K. Walsh, C. OʼRiordan, P. A. McCarthy, E. Rogers

**Affiliations:** ^1^ Department of Urology, University College Hospital, Galway, Ireland; ^2^ Department of Radiology, University College Hospital, Galway, Ireland

**Keywords:** renal, trauma, management, urologist

## Abstract

There is a paucity of data regarding renal trauma. The majority of cases of renal trauma are amenable to conservative management. We sought to streamline the management of renal trauma in the west of Ireland. Patients presenting with a computerised tomogram–confirmed renal injury were assessed over 5 years. Patient demographics, injury details, initial emergency department management, definitive management, and follow-up were assessed. Renal trauma was graded in a blind fashion (I-V). Twenty-five patients were identified; male:female (23:2). The mean age was 26 years. The majority of renal traumas were managed conservatively (92%); 8% patients underwent nephrectomy. The common mechanisms of renal injuries were road traffic accidents (44%). The majority of cases of renal injuries occur as a result of blunt trauma and can be conservatively treated. Two nephrectomies (8%) were performed. We believe this study potentially can be beneficial as part of an all-Ireland trauma database to improve patient outcome.

## INTRODUCTION

Renal trauma occurs in approximately 1–5% of all traumas[1]. The majority of renal injuries (90%) are due to blunt trauma causing direct damage (e.g., assault, sporting injuries, road accidents, or crush injuries) or indirect damage causing sudden displacement of the kidney (e.g., fall from a height)[1]. Penetrating injuries account for the remaining 10%, but given societal changes and increasing gun crime, this figure is set to increase. In earlier studies on renal trauma, the incidence of renal trauma was reported at 1.2 and 0.9%[2,3].

Traditionally, staging of renal injuries via clinical assessment, radiological imaging, and trauma scoring systems has permitted conservative management for the majority of blunt traumas. The traditional dictum of operating on all penetrating injuries currently remains controversial[1,3]. Definitive grading of renal injuries is by intravenous and oral contrast-enhanced computerised tomography (CT). CT is paramount when deciding which injuries can be managed expectantly, according to the American Society for the Surgery of Trauma (AAST), which has detailed five (I–V) grades of traumatic renal injuries. Grade I renal injury is defined as contusion or nonexpanding subcapsular haematoma without laceration. Grade II renal injury is defined as nonexpanding perirenal haematoma with cortical laceration <1 cm deep, but without urinary extravasation. Grade III renal injury is defined as cortical laceration >1 cm deep without urinary extravasation. Grade IV renal injury is defined as laceration through corticomedullary junction into the collecting system or vascular pedicle with contained haematoma. Grade V renal injury is defined as a shattered kidney, or vascular pedicle injury or avulsion[4]. The majority of blunt renal injuries can be managed conservatively and, indeed, there is evidence that some grade I and II penetrating renal injuries can be managed nonoperatively[5]. Indications for operative intervention include haemodynamic instability, associated life-threatening injuries, expanding retroperitoneal haematoma, and decreasing serial haemoglobin level[5,6].

Over 25 years ago, Archbold et al.[7] studied genitourinary injury patterns as a result of civilian violence in Northern Ireland. In 1991, Davies et al.[8] observed the surgical caseload of a district general hospital in the northeast of the republic of Ireland. The urological problems encountered in that study were 11% of the total surgical caseload[8]. Recently, one paper from the U.K. audited the volume of urological trauma, and its impact on resource and implications for the planning of trauma services[9]. In the republic of Ireland, a national trauma database has been recently established by the national neurosurgical centre at Beaumont Hospital, Dublin, Ireland, but no renal trauma data is included[10].

The aims of this study were to (a) assess the volume of upper tract renal trauma presenting to a university hospital in the west of Ireland, (b) analyse the modes in presentation (including mechanism of injury), and (c) comment on management patterns, including radiological methods employed, surgical intervention, and follow-up at out-patient level. This study could ultimately serve as a pilot study for a national database on renal trauma that guides urologists and resource trauma services nationally.

## PATIENTS AND METHODS

Between January 1, 2002 and June 31, 2007, all renal trauma patients presenting to a university hospital in the west of Ireland were identified using the Hospital In-Patients Enquiry (HIPE) system. The HIPE search terms employed were renal and trauma, and/or kidney and injury. Inclusion criteria were the following: all renal trauma patients presenting with a CT-confirmed renal injury (grade I–V) according to AAST. Exclusion criteria were (a) patients with negative findings of renal injury on CT following initial clinical suspicion and (b) patients initially managed at University College Hospital Galway (UCHG), but subsequently transferred to supraregional centres for specialist management following complex trauma to other organs, i.e., neurosurgical and cardiothoracic. Following initial resuscitation, two trauma patients were subsequently transferred to a specialist trauma centre for the management of severe neurosurgical and thoracic injuries, respectively. Although the renal injuries of these two patients were graded as I and II, respectively, they were excluded from this study due to the discontinuation of the management of their renal injuries at our institution following their transfer. A total of nine patients presented with clinical suspicion of renal trauma, but subsequently had negative CT findings and were thus excluded from this study. Although renal ultrasonography (US) was performed on six trauma patients with clinical suspicion of renal trauma, one patient had a positive finding of grade I renal injury. None of these renal injuries, however, were assessed or graded by CT.

A retrospective chart review was carried out using a proforma to assess (a) patient demographics, (b) details of the incident, (c) emergency department (ED) management, (d) radiological imaging, (e) specialist management, (f) laboratory parameters, (g) in-patient management (including immediate complications), and (h) out-patient follow-up (including delayed complications). We also examined the time required for (1) patients to reach the ED, (2) to involve urology or other medical/surgical specialties, and (3) time spent awaiting specialist imaging (CT) (Table 1).

Where applicable, we also examined for the presence of alcohol and illicit drug use. Injuries were anatomically classified according to the injury severity score (ISS)[11]. The physiological parameters were assessed with revised trauma score (RTS)[12]. Each renal injury detected on CT was reviewed in a blinded fashion by a consultant radiologist. These renal injuries were retrospectively graded (I–V) according to AAST guidelines[4].

**TABLE 1**
**Emergency Department Management of Trauma Patients**

| ED Management | G I = 5 | G II = 7 | G III = 7 | G IV = 5 | G V = 1 |
|---|---|---|---|---|---|
| Time taken to arrive to ED | 6.5 h | 7 h | 7 h | 3.25 h | 15 m |
| Time taken to involve urology | 5 h | 3.25 h | 4.2 h | 2.2 h | 10 m |
| Time taken to involve another specialty | 4.5 h | 4.25 h | 6.2 h | 3.2 h | 10 m |
| Time awaiting CT imaging postassessment | 16.5 h | 16.5 h | 6.5 h | 6.5 h | 30 m |

Values are represented by median figures; h, hour; m, minute.

The ISS is a scoring system derived from the abbreviated injury scale[11]. The ISS scores range from 0 to 75 (0 best to 75 unsurvivable), and correlate to injury, with minor injury represented by a score of <9, moderate injury by a score of 9–14, severe injury by a score of 15–24, and critical injury by a score of ≥25[11,12]. In 1989, this score was validated and devised by the AAST for individual organs, including kidneys, to facilitate clinical research[4].

The RTS was employed to assess patients' initial physiological parameters — including systolic blood pressure (BP, mmHg <90), respiratory rate (RR, <10 or ≥30), and Glasgow Coma Scale (GCS, <13) — and ranges from 0 to 12[12]. Blood transfusion requirements (units) and haemoglobin levels (Hb, g/dl) were also recorded in this study (Table 2). All injured patients had a physiological and haemodynamic assessment, together with a full blood count and renal biochemical profile, on admission.

**TABLE 2**
**Details of Operative Group**

| ED Management | G III = 1 | G V = 1 |
|---|---|---|
| Age (years old) | 24 | 25 |
| Sex | Female | Male |
| MOI | RTA | Sport related |
| Type of injury | Blunt | Blunt |
| Underlying renal pathology | None | None |
| SBP on arrival (mmHg) | 110 | 82 |
| GCS on arrival | 13 | 9 |
| Hb g/dl on arrival | 12.9 | 9.9 |
| RR/min on arrival | 22 | 25 |
| Blood transfusion | 4 | 10 |
| Associated injury | Hepatic lacerations | None |
| Nephrectomy | Delayed 9/7 | Immediate |

MOI, mechanism of injury; RTA, road traffic accident; SBP, systolic blood pressure; GCS, Glasgow Coma Scale; Hb, haemoglobin; RR, respiratory rate.

## RESULTS

Twenty-five patients with CT-graded renal trauma were identified during the study (23 males and two females). The mean age was 26 years (range: 7–59). Four patients (16%) were transferred from different regional institutions for further management. The majority of renal traumas (28%) were grade II and III (Table 3). Mechanisms of renal injuries were road traffic accidents (RTA) (11/25, 44%), sporting injuries (8/25, 32%), assault (3/25, 12%), fall (2/25, 8%), and stab injury (1/25, 4%). The incidence rate (IR) of renal trauma among the Irish population in the west of Ireland was calculated to be 10.25 cases per million person-years[1].

**TABLE 3**
**Renal Trauma Grading and Associated Injuries**

| Renal Grades | G I | G II | G III | G IV | G V |
|---|---|---|---|---|---|
| No. of patients | 5 | 7 | *7 | 5 | 1 |
| Conservative | 5 | 7 | 6 | 4 | — |
| Operative | — | — | 1 | — | 1 |
| Associated injury | — | 1 Pneumothorax, 2 rib fractures | 1 Hepatic injury | — | 1 Rib fracture |

* One patient was upgraded to grade III (sustained bilateral renal injury, grade II and grade I on left and right, respectively).

One trauma patient (4%) was critically injured (ISS ≥25) (immediate nephrectomy), two trauma patients (8%) were seriously injured (ISS 14–25), nine trauma patients (36%) sustained moderate injuries (ISS 9–14), and 13 trauma patients (52%) sustained minor injuries (ISS <9). Chest and abdominal injuries, such as pneumothorax and minor hepatic lacerations, were associated with the renal injuries. All of these associated injuries were managed conservatively and are listed in Table 3.

In this study, the details of management of renal trauma patients were observed. Time of patient transfer to the ED reduced dramatically from 6.5 h (grade I) to 15 min (grade V) as the renal injury increased. Regarding the time required to involve the urology team and other specialties, time was again reduced from 5 h (grade I) to 10 min (grade V) as the severity of renal injury increased. As expected, CT imaging was obtained in approximately 30 min after a patient's arrival to the ED in renal trauma grade V. However, trauma patients with grade I and II waited over 12 h for CT imaging, reflecting the severity of injury (Table 1).

The majority of renal trauma patients (92%) were managed conservatively; however, two patients (8%) underwent surgical intervention (nephrectomy). All nephrectomies were performed for blunt injuries: one was sports related and one was a result of RTA. Details of the operative group are listed in Table 2.

In the conservative group (n = 23), one patient sustained a sports-related grade II renal injury in the presence of known, underlying combined haematological and renal pathologies; Von Willebrand disease (vWD) and polycystic kidney disease (PCKD). Another patient in the conservative group who sustained a grade IV renal injury was found to have an underlying, previously undiagnosed pelviureteric junction obstruction (PUJO). A trauma-related haemorrhage in the underlying PUJO resulted in the formation of abscess in a poorly functioning hydronephrotic kidney, necessitating delayed nephrectomy 6 months following initial injury and treatment.

One penetrating injury due to a stab wound (grade III) was managed conservatively. All parameters remained normal throughout the in-patient management and the patient took his discharge against medical advice after 3 days and failed to attend for follow-up.

[1]This was derived using the Poisson distribution formula IR = C/ (P × T), where C is the cases observed during the period studied (25), P is the population at risk during the time observed (443,227), and T is the study time (5.5 years) = 25/(443,227 × 5.5). Assuming a 95% confidence interval (CI) (of 6.6 to 15.1 per million person-years), this gives a value of 0.000010255 or 10.25 cases per million person-years[13].

Immediate complications were noted in three patients, all with grade III injuries, including septicaemia, lower respiratory tract infection, and multiorgan failure (MOF). All of these complications were treated appropriately. Delayed complications were noted in five patients and included chronic pain (one patient with grade IV), renal scarring (one patient with grade III), hypertension (one patient with grade II injury), post-traumatic stress syndrome (PTSS) (one patient with grade III injury), and poorly functioning hydronephrotic kidney (one patient with grade IV injury). There was no mortality associated with any of the renal injuries studied and/or with other associated injuries during the period observed.

Three months following the renal injury, radiological CT follow-up was performed on 8/25 patients (32%) (two, three, and three patients sustained grade II, III, and IV renal injuries, respectively). Although CT confirmed resolution of haematoma, paranchymal, and/or urinary injuries, one patient with grade III renal injury developed minor renal scarring on CT imaging postinjury. No renal imaging was performed on the remaining renal trauma patients (13/25, 52%) in the postinjury period. Four out of the 25 renal trauma patients (16%) with grade I (three patients) and grade II (one patient) renal injuries failed to attend their radiological follow-up appointments.

The association between grade of renal injuries and the presence of either alcohol and/or substance abuse was also examined. No patients with higher-grade renal injuries (IV and V) were found to be alcohol and/or substance intoxicated. However, patients sustaining less severe renal injuries were found to have used alcohol and illicit substances. Alcohol use was the highest among patients with grade II renal injury (three patients, 60%).

## DISCUSSION

Renal trauma is an important, but poorly defined, contributor to the urological workload in the west of Ireland. It may occur as an isolated event or may involve multiple surgical teams in the polytrauma setting. To date, there have been very few studies on upper tract trauma in the Irish setting, particularly in the early 1980s and 1990s[7,8]. In this study, we have shown that the majority of cases, including selective stable penetrating injuries, are amenable to expectant management.

Based on 2006 population figures, there were 443,227 persons living within the western region, which includes Galway, Mayo, and Roscommon[14]. The incidence rate (IR) of renal trauma among the Irish population in the west of Ireland was calculated to be 10.25 cases per million person-years. In the U.K., the incidence of renal injury is reported to be about 50 cases per million person-years[15].

In the past decade, there has been a sharp increase in gun-related crime in several major urban regions in the republic of Ireland[14]. Interestingly, during the period observed in this study, there were no gunshot injuries recorded in the western area. It is known that penetrating renal trauma, most commonly as a result of gunshot and stab wounds, has an increased incidence of major renal injury and associated intra-abdominal injury, which often requires operative intervention[16].

In a recent consensus on the management of renal trauma, bilateral renal injury can be advanced to grade III injury[17]. In our study, similar findings were observed in which one patient sustained bilateral renal injury (grade II and grade I on left and right, respectively) and was subsequently upgraded to grade III (Table 3).

In patients with higher-grade renal injuries, time spent waiting for CT imaging was less than 1 h. In one case, a patient with a grade V injury was haemodynamically unstable on presentation, necessitating immediate nephrectomy. This patient was transferred to the ED in approximately 15 min and taken to theatre less than 1 h after CT imaging. Not surprisingly, patients who sustained higher renal injuries (grade III, IV, and V) presented with deranged RTS, low haemoglobin, and required transfusion (Table 2). Although thoracic and abdominal injuries were observed in 5/25 (20%) trauma patients with grade II, III, and V renal injuries, only 1/25 (4%) patients with grade III renal injury required nephrectomy (Tables 2 and 3). Length of hospitalisation varied according to the severity of the renal injury. It was noted that the higher the renal injury, the greater the hospital stay. Our findings were consistent with larger published studies regarding management of renal trauma[18].

CT is considered the gold standard imaging modality of renal trauma[17,20]. In our study, six patients with clinical suspicion of renal trauma were imaged by renal US. Moreover, none of these patients underwent subsequent CT imaging. In terms of radiological follow-up, there is no strong evidence in the published literature to support imaging post–renal injuries. However, Blankenship et al.[19] highlighted the importance of delayed imaging, particularly in the immediate postinjury period, in order to identify delayed complications that may require future intervention. In our study, CT was the modality of choice for radiological follow-up post–renal injury. Only 8/25 patients (32%) with grade II–IV renal injuries had CT follow-up approximately 3 months postinjury. Four patients (16%) with grade I, II, and III renal injuries failed to attend the out-patient urology clinic, while the majority of patients were regularly followed-up in the out-patient clinic. This study demonstrates some shortfalls in the current renal trauma protocol, particularly in its radiological aspects. Such potential weaknesses can be avoided by implementing and adhering to the guidelines published by the AAST[4].

The nephrectomy rate was 8%, and the overall complication rate was low and in keeping with larger international series[20]. Although there was no mortality associated with any of the renal injuries, there were immediate and delayed complications in three and five patients, respectively, which were treated as required.

A recent Irish study on trauma from the national neurosurgical centre has highlighted the extent of severe injuries attributed to RTA (34%) and, in addition, has acknowledged the increasing number of injuries secondary to assault[10]. However, in the west of Ireland, the common mechanisms of renal injuries were RTA (11/25, 44%) and sporting injuries (8/25, 32%).

The establishment of national renal trauma guidelines are necessary as trauma should ideally be managed in centres of expertise, with adequate resources, be protocol driven, and subject to continuous audit. If established, this would allow comparison with European trauma data and further enhance outcomes of renal trauma in Ireland. At the hospital level, administrative officers and executives should be made aware of such important clinical data in order to justify having a trauma database and to further advance the trauma centres at their institutions. With the current restructuring of the Irish Health Service and the creation of major centres specialising in multidisciplinary management of trauma, it is important to acknowledge the role of the urologist in the management of trauma patients.

In conclusion, this study is the first report on renal trauma in Ireland that can continuously and progressively enrich a larger pool of trauma data, and ultimately help to form an all-Ireland trauma database that should aid patients' outcomes and promote "best practice" in managing renal trauma patients.
